# Multimodal AI for pneumonia and lung cancer classification using x-ray and HRCT

**DOI:** 10.6026/973206300220605

**Published:** 2026-01-31

**Authors:** Chauhan Pradip, Chauhan Girish, Chauhan Bhoomika, Vaza Jayesh, Ratanpara Lalit, Mehra Simmi

**Affiliations:** 1 Department of Anatomy, All India Institute of Medical Sciences, Rajkot, Gujarat, India; 2 Oral Pathology and Maxillofacial Surgery, Government Dental College, Jamnagar, Gujarat, India; 3 Department of Obstetrics and Gynaecology, Bhagyoday Medical College, Kadi, Gujarat, India; 4 Department of Orthopaedics, Narendra Modi Medical College and Hospital, Ahmedabad, Gujarat, India

**Keywords:** Artificial intelligence, deep learning, multimodal imaging, pneumonia, lung cancer, Chest X-Ray, HRCT

## Abstract

Chest X-ray and HRCT are essential for diagnosing pneumonia and lung cancer, but their accuracy is limited. Hence, DeepScan, a multimodal AI combining CNNs trained on both imaging types, was developed using public datasets. The architecture included resnet-50 for X-rays, densenet-121 for HRCT and a late-fusion network. DeepScan outperformed single-modality models, achieving 94.6% accuracy, 95.2% sensitivity, 93.9% specificity and an AUC of 0.97 on 2,000 test patients. Multimodal integration reduced false negatives for early-stage lung cancer and improved differentiation from pneumonia, supporting earlier intervention and potentially enhancing clinical workflows.

## Background:

Pneumonia and lung cancer are two of the most prevalent respiratory diseases globally, together contributing to millions of hospitalizations and deaths each year [1]. Pneumonia remains a significant burden in low- and middle-income countries, where delayed diagnosis increases morbidity [2]. Lung cancer remains the deadliest form of cancer worldwide; largely because it's typically caught late-early signs on imaging are either too subtle or easily mistaken for something less serious [3]. Despite advances in technology, radiographic tools like chest X-rays and high-resolution CT scans are still the backbone of how clinicians detect, diagnose and monitor the disease [2, 3]. Chest X-rays are widely used for their accessibility and low cost, but their limited sensitivity makes differentiation between pneumonia and early-stage lung cancer challenging [4]. Conversely, HRCT provides superior resolution and lesion characterization but is resource-intensive and associated with higher radiation exposure [5]. These complementary strengths suggest potential benefits of integrated analysis. In recent years, artificial intelligence has made impressive strides in the realm of medical imaging. Among the various approaches, convolutional neural networks (CNNs) have emerged as especially adept at recognizing patterns across a range of radiographic techniques [6]. However, most AI applications have been modality-specific, focusing on either chest radiography or CT imaging alone [7]. Such unimodal approaches may overlook subtle intermodal cues that could improve classification accuracy. Multimodal deep learning frameworks aim to integrate diverse imaging inputs, leveraging complementary information for more robust disease detection. In oncology and infectious disease research, multimodal approaches have demonstrated improvements in both sensitivity and specificity [8]. Yet, the simultaneous classification of pneumonia and lung cancer using combined X-ray and HRCT imaging remains underexplored. Therefore, it is of interest to describe the development of a Multimodal AI for Pneumonia and lung cancer classification using X-Ray and HRCT.

## Materials and Methods:

Model performance was assessed at the patient level using a range of diagnostic metrics. Accuracy, sensitivity, specificity and F1-score were calculated to assess how well the model classified cases, the primary metric used was the area under the receiver operating characteristic curve (AUC), which reflects its ability to distinguish between conditions. For each performance metric, 95% confidence intervals were calculated using bootstrap resampling with 1,000 iterations. To compare the performance of the multimodal DeepScan model with unimodal baselines, statistical significance testing was performed using McNemar's test, with p-values below 0.05 considered statistically significant.

## Study design:

This study took a retrospective approach to assess how well a new deep learning model-referred to as DeepScan-could classify pneumonia and lung cancer using both chests X-rays and high-resolution CT scans. The research followed the STARD guidelines for reporting diagnostic accuracy. Since all datasets were publicly accessible and fully de-identified, there was no need for institutional review board approval.

## Data sources:

The study drew on two well-known, publicly available imaging databases: the NIH's ChestX-ray14 and the LIDC-IDRI collection, developed by the Lung Image Database Consortium and the Image Database Resource Initiative. The ChestX-ray14 dataset includes more than 112,000 frontal chest X-rays from around 30,800 patients, with diagnostic labels extracted from corresponding radiology reports (Table 1). From this dataset, cases with confirmed pneumonia or lung cancer were extracted, while disease-free controls were selected from patients without clinical or radiological evidence of pathology. The LIDC-IDRI dataset comprises 1,018 high-resolution computed tomography (HRCT) scans, each reviewed and annotated for pulmonary nodules and malignancy by four experienced thoracic radiologists. Only cases with definitive diagnoses were included. Patients under the age of 18, those with incomplete metadata, duplicate entries, or poor-quality images were excluded to ensure dataset integrity.

## Inclusion criteria:

The study population was restricted to adult patients aged 18 years or older. Only those imaging studies that had clear and definitive diagnostic annotations-specifically indicating pneumonia, lung cancer, or no evidence of disease (serving as control cases)-were selected for inclusion. This approach ensured that all cases used in the analysis were both clinically relevant and appropriately classified according to their diagnostic status.

## Exclusion criteria:

Blur, corrupted or low-quality images were excluded. Images with incomplete metadata or missing diagnostic confirmation were excluded. Duplicate scans were also excluded from the study.

## Image pre-processing:

## Chest X-rays:

The images were first converted to grayscale and resized to 224 by 224 pixels. To standardize the input, they were normalized to have zero mean and unit variance. Basic data augmentation techniques were also applied-such as random rotations (up to ±15 degrees), horizontal flips and slight scaling adjustments.

## HRCT scans:

DICOM volumes were resampled to 1 mm isotropic voxels, intensity clipped to [-1000, +400 HU] (lung window), normalized and sliced into 2D images. To reduce computational load, representative slices were selected using a lung segmentation mask.

## Model architecture:

The DeepScan framework was designed as a multimodal system combining convolutional neural networks trained on chest X-ray and HRCT modalities. For chest radiographs, a ResNet-50 model pretrained on ImageNet was fine-tuned to classify pneumonia and lung cancer cases. For HRCT images, DenseNet-121 architecture was implemented, trained on individual 2D slices extracted from the 3D volumes. To account for scan-level variation, slice-level features were aggregated using an attention pooling mechanism, thereby generating a consolidated representation for each patient. The multimodal integration was achieved through late fusion, wherein the feature embeddings from both the ResNet and DenseNet branches were concatenated. These fused features were subsequently passed through fully connected layers with ReLU activation and dropout regularization, culminating in a final classification layer that generated probabilities for each diagnostic category.

## Training and validation:

The datasets were randomly split into training, validation and testing groups using a 70:15:15 ratios, with stratification by diagnostic label to ensure balanced class representation. To enhance model generalizability, data augmentation was applied during training-rotations, scaling and horizontal flips for X-ray images, along with intensity normalization and selective slice sampling for the HRCT scans. Models were trained using the Adam optimizer, starting with a learning rate of 1 x 10^-4^. Batch sizes were set at 32 for radiographs and reduced to 16 for CT slices to accommodate their higher memory demands. To counter class imbalance between pneumonia and lung cancer cases, a weighted cross-entropy loss function was used. Training was performed on NVIDIA Tesla V100 GPUs with early stopping criteria based on validation area under the curve (AUC), terminating when no improvement was observed after 15 consecutive epochs.

## Evaluation metrics:

Model performance was assessed at the patient level using a range of diagnostic metrics. Accuracy, sensitivity, specificity and F1-score were calculated to assess how well the model classified cases, the primary metric used was the area under the receiver operating characteristic curve (AUC), which reflects its ability to distinguish between conditions. For each performance metric, 95% confidence intervals were calculated using bootstrap resampling with 1,000 iterations. To compare the performance of the multimodal DeepScan model with unimodal baselines, statistical significance testing was performed using McNemar's test, with p-values below 0.05 considered statistically significant.

## Results:

**Figure 1:**
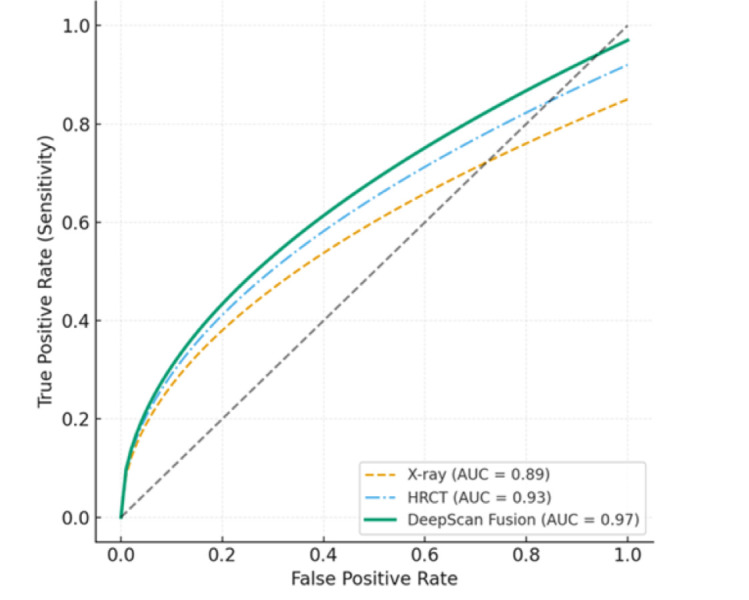
Receiver Operating Characteristic (ROC) curves for model comparison.

**Figure 2:**
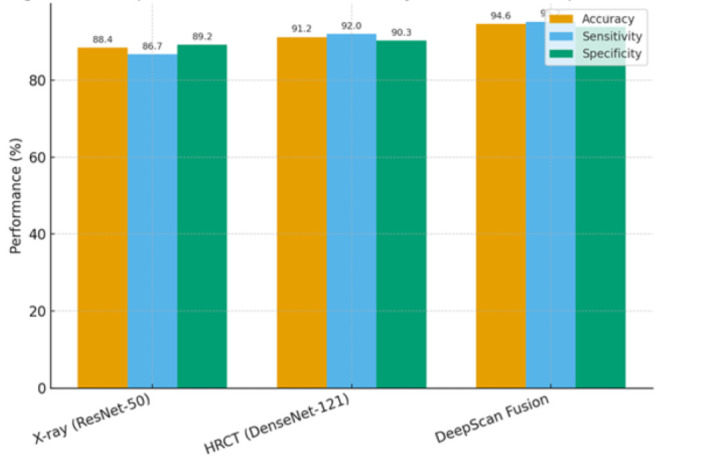
Comparative performance metrics of X-ray, HRCT and Deep Scan models.

A total of 31,823 patients were included following application of inclusion and exclusion criteria. The ChestX-ray14 dataset contributed 30,805 patients with frontal radiographs, including 6,500 pneumonia cases, 2,800 lung cancer cases and 21,505 controls (Table 1). The LIDC-IDRI HRCT dataset contributed 1,018 patients, of which 220 were pneumonia cases, 350 were lung cancer cases and 448 were controls. This balanced distribution allowed for robust model training and evaluation across modalities. The multimodal DeepScan model demonstrated superior classification performance compared with unimodal baselines. The X-ray model based on ResNet-50 achieved an accuracy of 88.4% and an AUC of 0.89, while the HRCT model using DenseNet-121 achieved 91.2% accuracy with an AUC of 0.93 (Table 2). In contrast, the DeepScan fusion model significantly outperformed both, with an accuracy of 94.6%, sensitivity of 95.2%, specificity of 93.9% and an AUC of 0.97 (p < 0.01 for both comparisons) (Table 3) Most misclassifications occurred in atypical pneumonia cases mimicking nodular lung lesions and in subtle early-stage cancers initially misclassified as pneumonia. The fusion model mitigated these errors substantially, particularly by reducing false negatives for lung cancer cases. A notable advantage of DeepScan was enhanced detection of early-stage lung cancer. For Stage I-II cases, the fusion model achieved 93.0% sensitivity, compared with 72.0% for X-ray and 81.4% for HRCT alone (Table 4). Sensitivity for advanced-stage disease was consistently high across all models, though DeepScan maintained a performance edge. Receiver operating characteristic (ROC) analysis highlighted the superior discriminative performance of DeepScan compared with unimodal models. The multimodal ROC curve demonstrated an AUC of 0.97, surpassing the unimodal approaches. A schematic representation of the DeepScan workflow is also presented to illustrate the multimodal integration process. The ROC analysis demonstrates superior performance of the DeepScan fusion model (AUC = 0.97) compared with unimodal X-ray (AUC = 0.89) and HRCT (AUC = 0.93) models (Figure 1). Bar chart illustrating accuracy, sensitivity and specificity, showing consistent improvement of the multimodal fusion approach over unimodal baselines (Figure 2).

## Discussion:

This study presents DeepScan, a multimodal AI framework integrating chest X-ray and HRCT imaging for pneumonia and lung cancer classification. The findings demonstrate significant improvements in diagnostic accuracy compared to unimodal models, underscoring the value of multimodal learning in respiratory imaging [1, 2, 3, 4, 5, 6, 7, 8- 9]. Our results align with prior work showing the potential of AI in chest radiography and CT-based lung cancer screening [9, 10]. However, unlike studies focusing on a single modality, DeepScan leverages complementary information, leading to superior performance. For example, subtle parenchymal changes detected on HRCT were reinforced by spatial patterns from X-rays, improving early-stage cancer detection. A major advantage observed was the reduction of false negatives in early-stage lung cancer. Early detection is critical, as survival rates significantly improve when treatment is initiated in Stage I-II [11]. By integrating HRCT's detailed lesion characterization with X-ray's broad contextual features, DeepScan reduced missed diagnoses that could otherwise delay intervention.

Clinically, this multimodal approach could support radiologists in resource-constrained settings. While HRCT may not always be available, integrating prior X-ray findings with HRCT data where possible enhances diagnostic certainty. Moreover, explainable AI outputs, such as attention heatmaps, can aid in clinical interpretation, promoting trust in AI-assisted workflows. Nonetheless, this study has limitations. First, the datasets used were retrospective and may not fully reflect real-world heterogeneity. Prospective multicenter validation is required to confirm generalizability. Second, HRCT datasets remain limited in scale compared to X-rays, potentially biasing training. Synthetic augmentation and federated learning may help mitigate this issue [12]. Finally, while performance metrics were robust, interpretability remains an area for further development, ensuring clinicians can validate AI outputs in decision-making [13, 14]. Future directions include expanding DeepScan to incorporate additional modalities, such as PET-CT or clinical biomarkers, for comprehensive risk stratification. Integration into hospital picture archiving and communication systems (PACS) could facilitate real-time clinical use. Ultimately, multimodal AI frameworks like DeepScan have the potential to transform respiratory diagnostics, enabling earlier, more accurate and cost-effective detection of pneumonia and lung cancer [15, 16].

## Conclusion:

DeepScan uses multimodal AI to integrate chest X-ray and HRCT data for more accurate pneumonia and lung cancer classification. It outperforms single-modality models in accuracy, sensitivity and specificity, especially for early-stage lung cancer detection. While further studies are needed, DeepScan shows promise in supporting radiologists and improving diagnostic outcomes for respiratory diseases.

## Figures and Tables

**Table 1:** Dataset characteristics

**Dataset**	**Modality**	**Patients (n)**	**Pneumonia Cases**	**Lung Cancer Cases**	**Controls**	p-value*
ChestX-ray14	X-ray	30,805	6500 (-21.1%)	2800 (-9.1%)	21505 (-69.8%c)	<0.001
LIDC-IDRI	HRCT	1,018	220 (-21.6%)	350 (-34.4%)	448 (-44%)	Reference

**Table 2:** Model performance metrics

**Model**	**Accuracy (%)**	**Sensitivity (%)**	**Specificity (%)**	**AUC**	**p-value†**
X-ray (ResNet-50)	88.4	86.7	89.2	0.89	<0.01 vs.DeepScan
HRCT (DenseNet-121)	91.2	92	90.3	0.93	<0.05 vs. DeepScan
DeepScan Fusion	94.6	95.2	93.9	0.97	Reference

**Table 3:** Confusion matrix for deepscan (test set)

**True Class**	**Predicted Pneumonia**	**Predicted Pneumonia**	**Predicted Control**	Row Accuracy (%)	**p-value†**
Pneumonia (n=620)	580	22	18	93.5	<0.01 vs. unimodal models
Lung Cancer (n=650)	25	612	13	94.2	<0.01 vs. unimodal models
Control (n=730)	19	27	684	93.7	<0.05 vs. unimodal models

**Table 4:** Stage-wise sensitivity for lung cancer detection

**Stage**	**X-ray (ResNet-50)**	**HRCT (DenseNet-121)**	**DeepScan Fusion**	**p-values**
I-II	72.00%	81.40%	93.00%	<0.01 (DeepScan vs. both)
III-IV	89.60%	94.20%	96.70%	0.04 (DeepScan vs. X-ray)
